# Supplementary material for: Oxidative stress-induced MMP- and γ-secretase-dependent VE-cadherin processing is modulated by the proteasome and BMP9/10
Source: Sci Rep. 2023 Jan 11;13:597. doi: 10.1038/s41598-022-27308-2 (PMC9834263; doi:10.1038/s41598-022-27308-2)
Supplement: Supplementary file 1 — Supplementary Information. [file 41598_2022_27308_MOESM1_ESM.pdf]

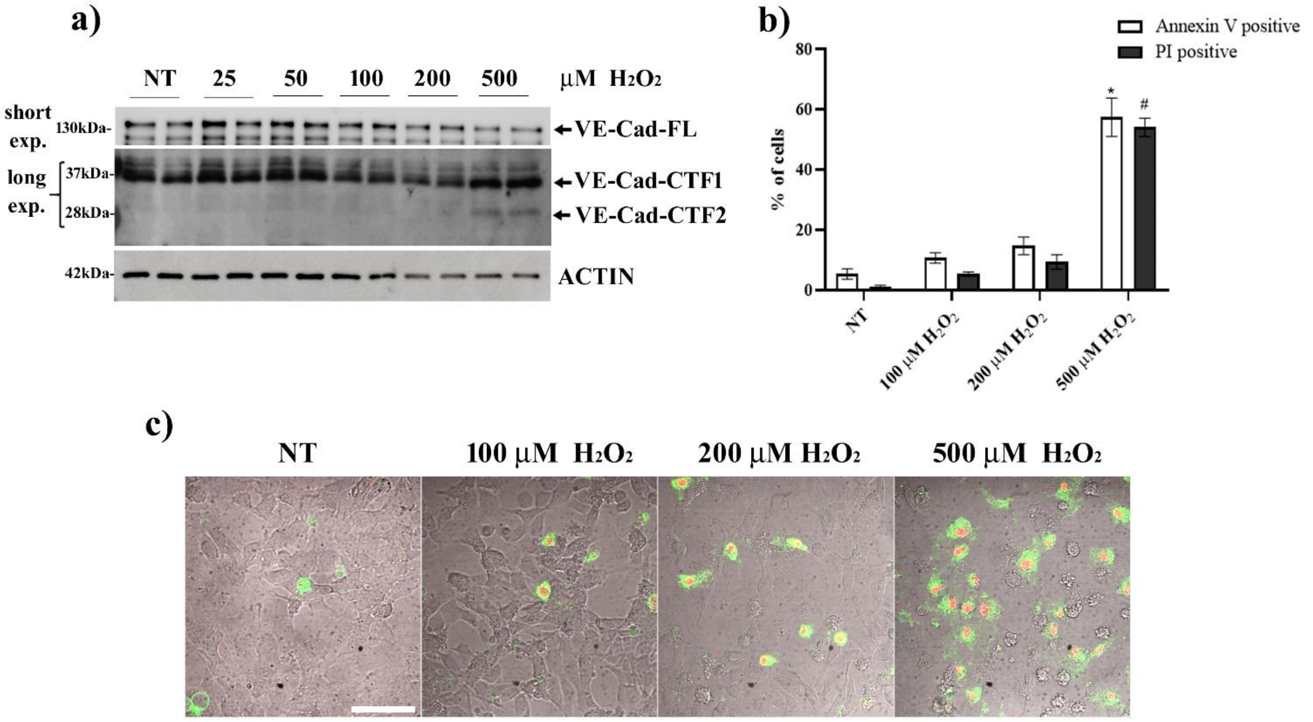

Supplementary Fig.1

Supplementary Fig.1: VE-Cad/CTF2 generation and apoptosis induction in HUVECs exposed to increasing concentration of H_2O_2. a) WB analysis of VE-cadherin in HUVECs treated with increasing concentration of H_2O_2, as indicated. Actin was used as loading control. Results are representative of 2 independent experiments. b) Percentage of Annexin V FITC positive cells and propidium iodide (PI) positive cells was calculated counting 4 fields for each experimental condition in 3 independent experiments. Data are mean ± s.e.m, *P<0.001 vs AnnexV FITC positive cells in NT, 100 µM and 200 µM H_2O_2, # P<0.001 vs PI positive cells in NT, 100 µM and 200 µM H_2O_2. c) Representative images of HUVECs exposed to increasing concentrations of H_2O_2 and stained with annexin V-FITC/PI, as described in materials and methods. Scale bar=50 µm.

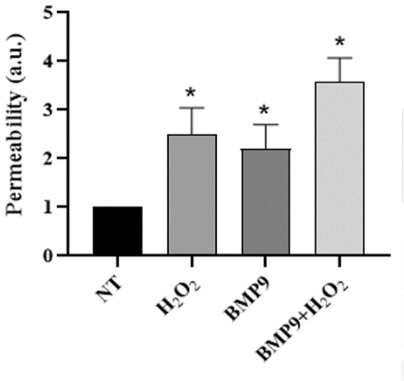

Supplementary Fig.2

Supplementary Fig.2: BMP9 does not prevent oxidative stress-induced permeability. The graph shows permeability of HUVECs pre-treated with BMP9 and exposed to 500 µM H_2O_2 for 6 hours. Data are mean ± s.e.m. of 3 independent experiments. *P<0.01 vs NT.

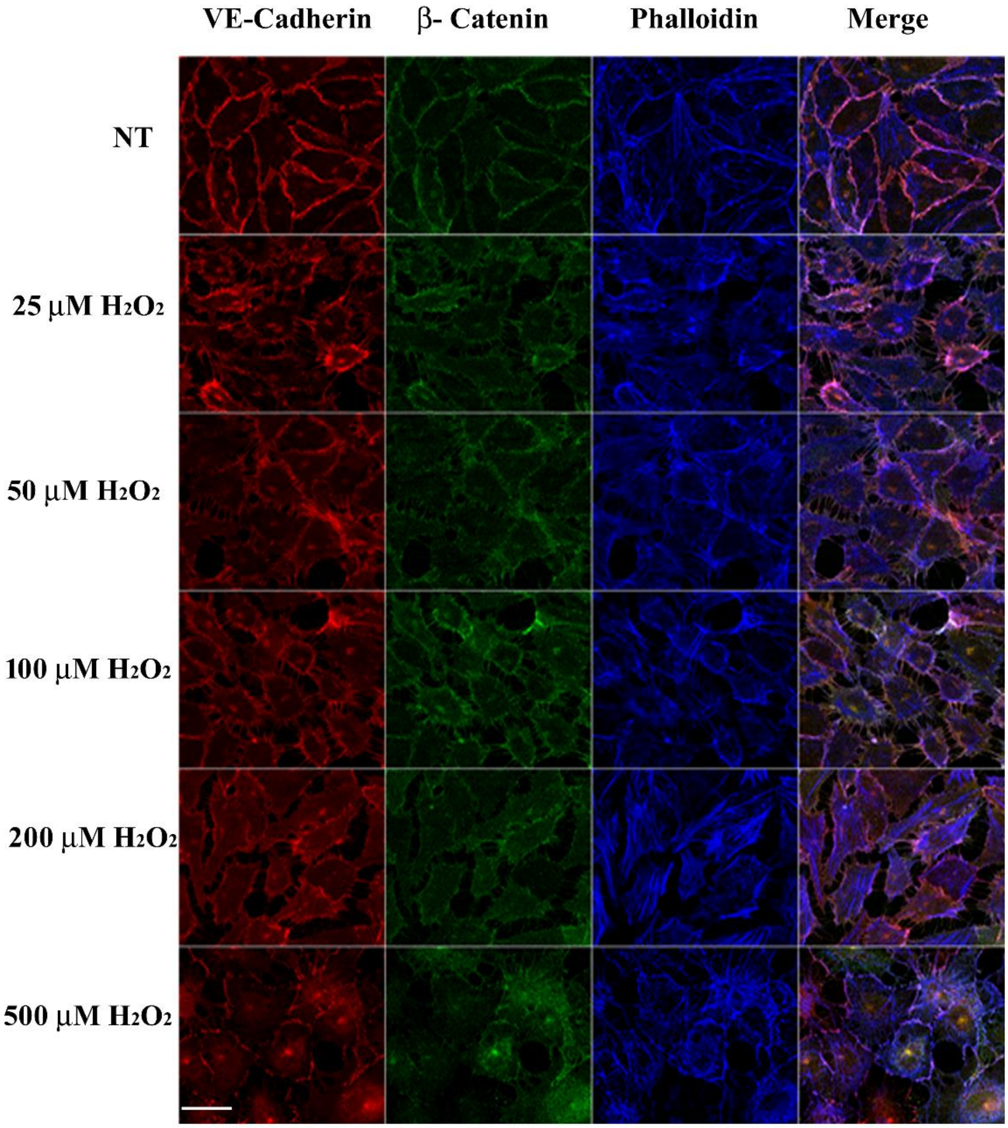

Supplementary Fig.3

Supplementary Fig.3: VE-cadherin, β-catenin, and F-actin distribution in HUVECs exposed to increasing concentration of H_2O_2. HUVECs were exposed to H_2O_2 (25-500 µM) for 6h. HUVECs were stained for VE-Cadherin (red), β-Catenin (green) using specific antibodies. Phalloidin was used to stain F-actin (blue). Scale bar=30 µm.

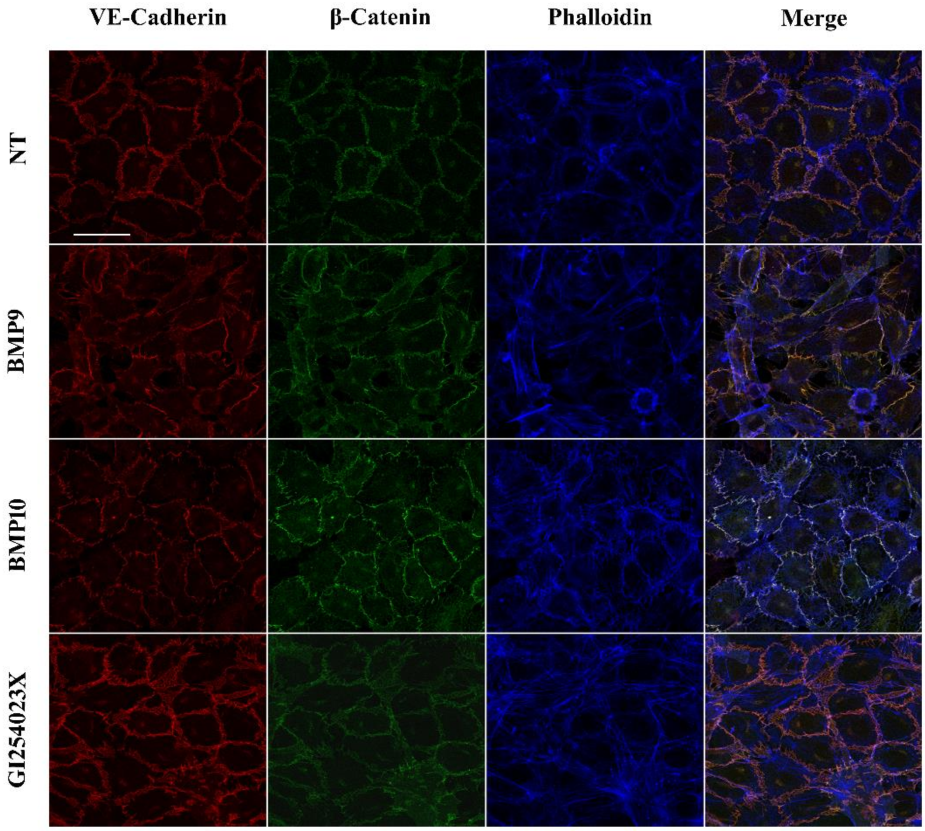

Supplementary Fig.4

Supplementary Fig.4: VE-cadherin, β-catenin, and F-actin distribution in HUVECs exposed to BMP9/10 or MMPs inhibitor. HUVECs were pre-treated with BMP9 (10 ng/ml), BMP10 (10 ng/ml) or GI254023X (10 µM) for 24h. HUVECs were stained for VE-Cadherin (red), β-Catenin (green) using specific antibodies. Phalloidin was used to stain F-actin (blue). Scale bar=30 µm. The panels are from the same representative experiment shown in Fig.4.

SHORTER EXPOSURE BLOTS USED FOR
FULL LENGTH

E-Cad/FL

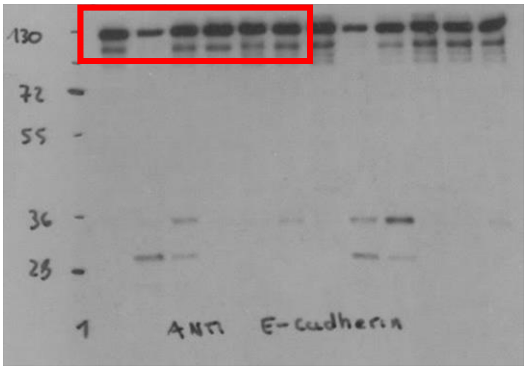

VE-Cad/FL

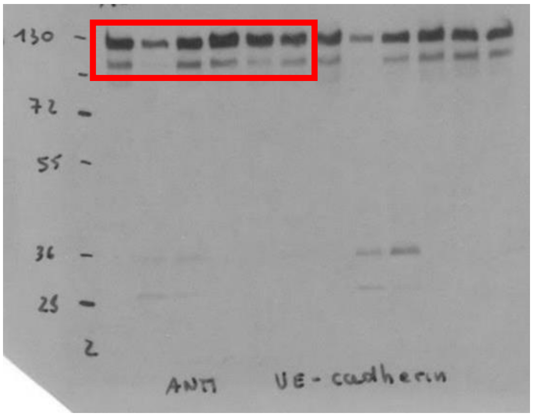

LONGER EXPOSURES USED FOR CTF1 and CTF2

E-Cad/CTF1 and
E-Cad/CTF2

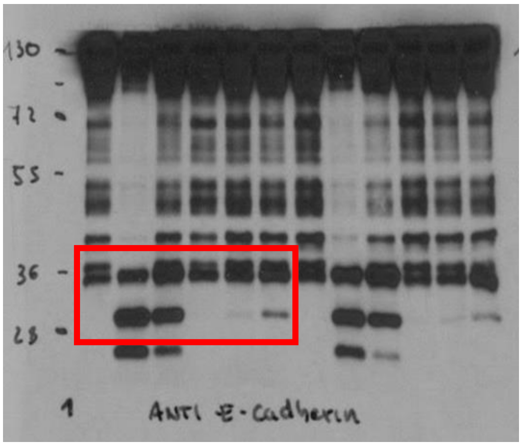

VE-Cad/CTF1 and
VE-Cad/CTF2

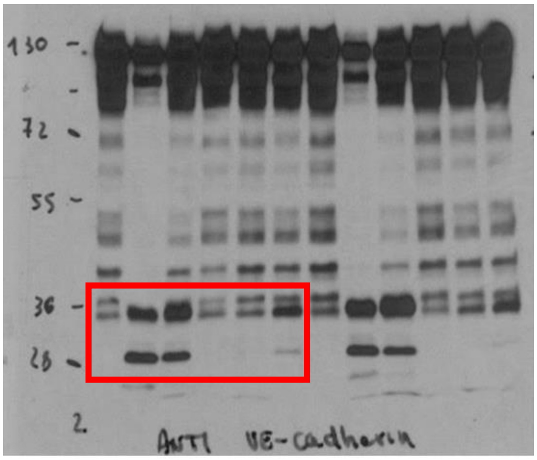

Fig.1

SHORTER EXPOSURE BLOTS USED FOR
VE-cadherin FULL LENGTH, and ACTIN

VE-Cad/FL

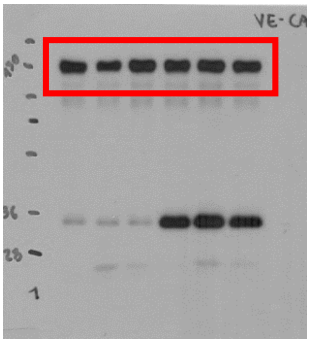

ACTIN

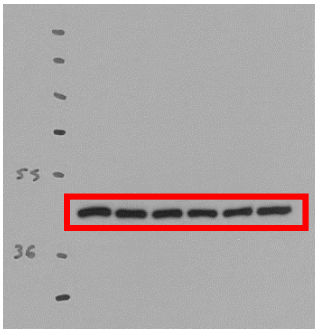

LONGER EXPOSURE USED FOR VE-Cad/CTF1
AND VE-Cad/CTF2

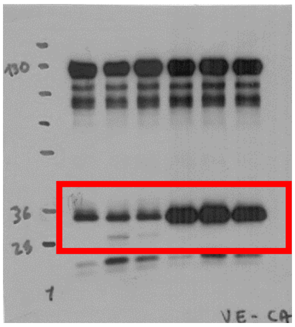

VE-Cad/CTF1
VE-Cad/CTF2

MUCH LONGER EXPOSURE

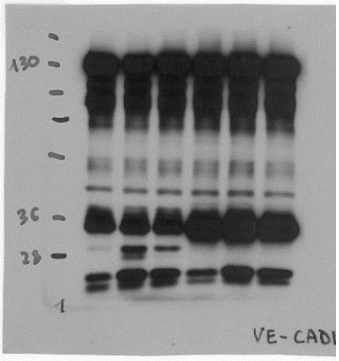

VE-Cad/CTF2

Fig. 2a

SHORTER EXPOSURE USED FOR VE-cadherin FULL LENGTH

VE-Cad/FL

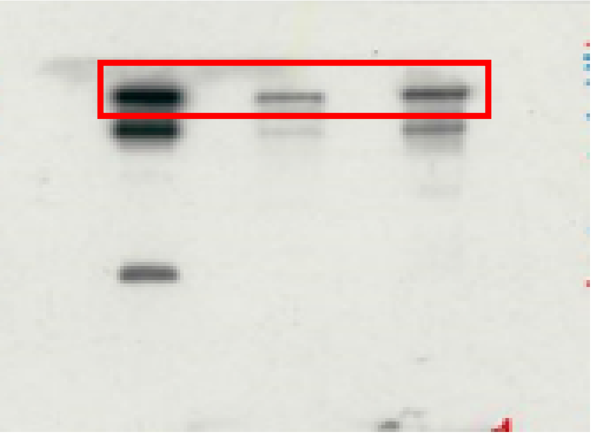

LONGER EXPOSURE USED FOR VE-Cad/CTF1 AND VE-Cad/CTF2

VE-Cad/CTF1
and VE-Cad/CTF2

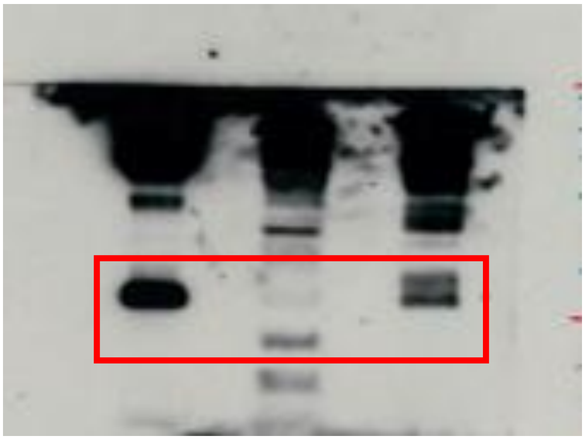

Fig. 2b

SHORTER EXPOSURES USED FOR
VE-cadherin FULL LENGTH, ACTIN AND P-smad1,5,8

VE-Cad/FL

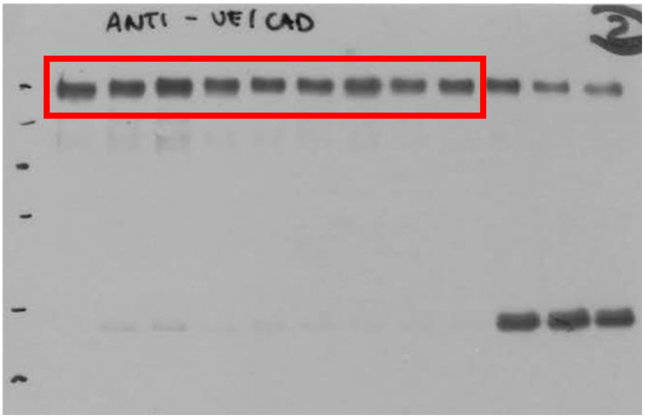

ACTIN

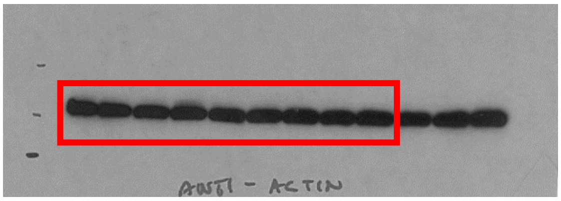

P-smad 1,5,8

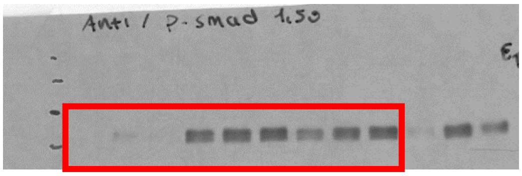

LONGER EXPOSURE USED FOR VE-Cad/CTF1 and VE-Cad/CTF2

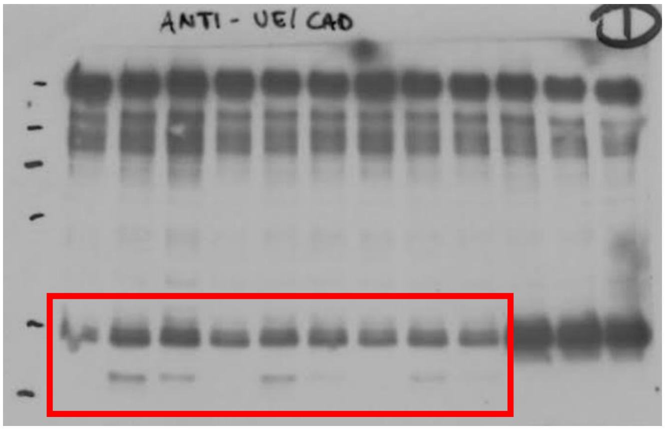

VE-Cad/CTF1

VE-Cad/CTF2

Fig. 2c

SHORTER EXPOSURE BLOTS USED FOR
VE-cadherin FULL LENGTH and ACTIN

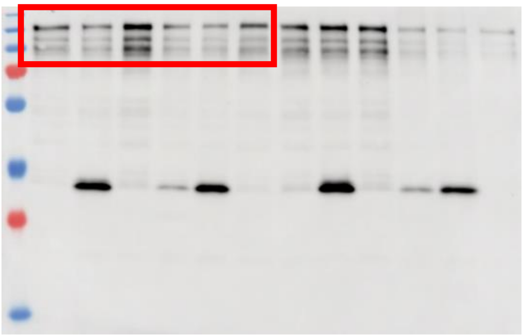

VE-Cad/FL

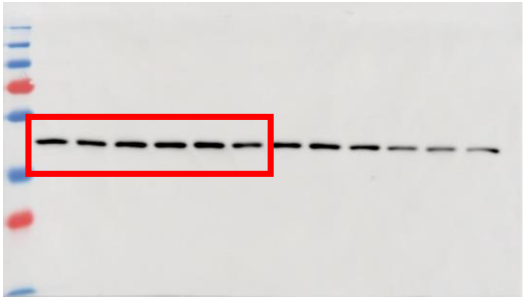

ACTIN

LONGER EXPOSURE BLOT USED FOR
VE-cadherin CTF1 and CTF2

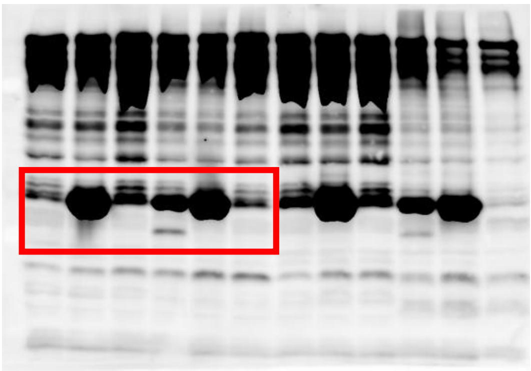

VE-Cad/CTF1
VE-Cad/CTF2

Fig. 3a

SHORTER EXPOSURE BLOTS USED FOR
VE-cadherin FULL LENGTH, ACTIN AND P-smad1,5,8

VE-Cad/FL

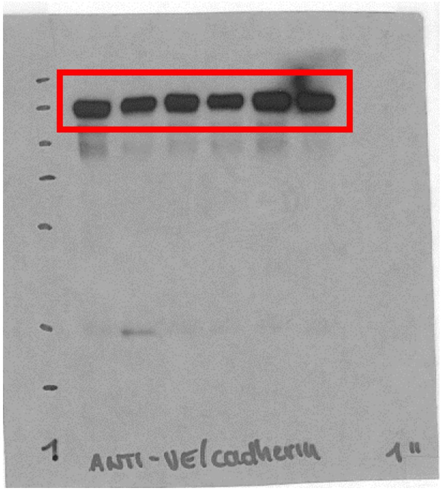

ACTIN

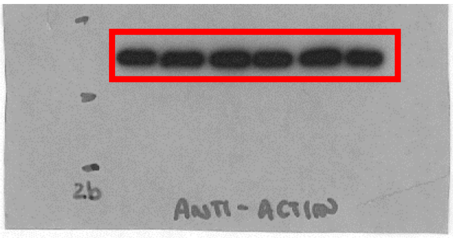

P-smad 1,5,8

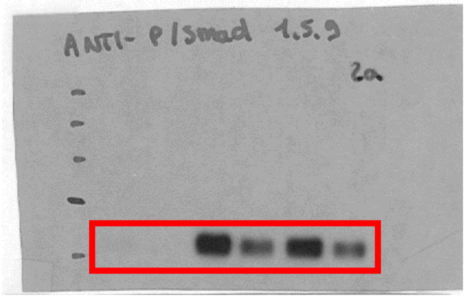

P-JNK

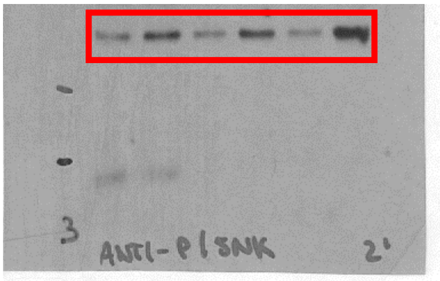

LONGER EXPOSURE USED FOR VE-Cad/CTF1 and CTF2

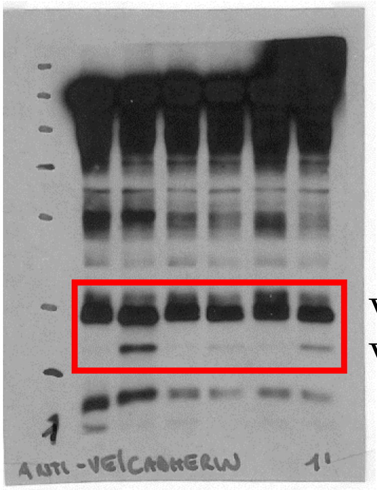

VE-Cad/CTF1
VE-Cad/CTF2

Fig. 3b

SHORTER EXPOSURE BLOTS USED FOR
VE-cadherin FULL LENGTH and ACTIN

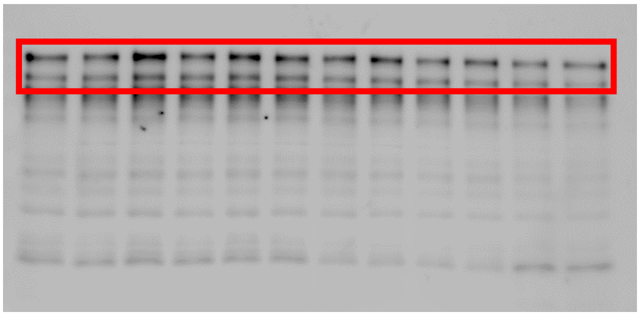

VE-Cad/FL

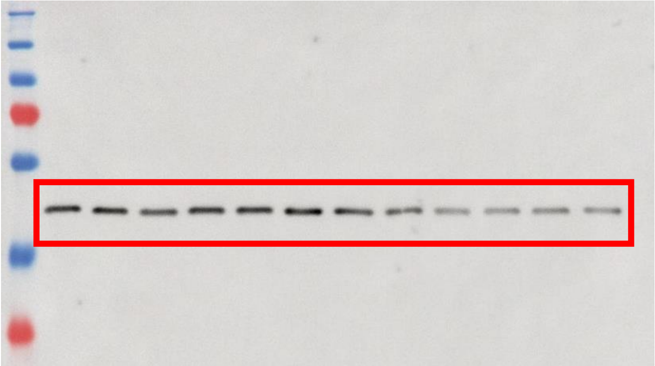

ACTIN

LONGER EXPOSURE USED FOR VE-Cad/CTF1 and CTF2

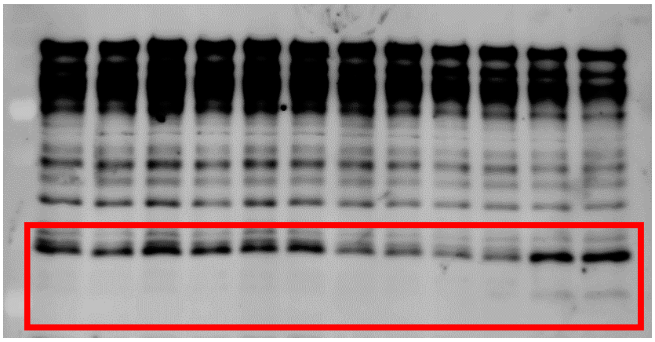

VE-Cad/CTF1
VE-Cad/CTF2

Suppl Fig. 1a
